# Cell-type dependent regulation of pluripotency and chromatin remodeling genes by hydralazine

**DOI:** 10.1186/s13287-023-03268-w

**Published:** 2023-03-16

**Authors:** Alain Aguirre-Vázquez, Fabiola Castorena-Torres, Beatriz Silva-Ramírez, Katia Peñuelas-Urquides, María Elena Camacho-Moll, Luis A. Salazar-Olivo, Iván Velasco, Mario Bermúdez de León

**Affiliations:** 1 Departamento de Biología Molecular, Centro de Investigación Biomédica del Noreste, Instituto Mexicano del Seguro Social, 64720 Monterrey, Nuevo León Mexico; 2 Depto. de Biología Molecular, Instituto Potosino de Investigación Científica y Tecnológica, 78216 San Luis Potosí, S.L.P. Mexico; 3 Escuela de Medicina, Tecnologico de Monterrey, 64710 Monterrey, Nuevo León Mexico; 4 Departamento de Inmunogenética, Centro de Investigación Biomédica del Noreste, Instituto Mexicano del Seguro Social, 64720 Monterrey, Nuevo León Mexico; 5 Instituto de Fisiología Celular-Neurociencias, Universidad Nacional Autónoma de México, 04510 Mexico City, Mexico; 6 Laboratorio de Reprogramación Celular, Instituto Nacional de Neurología y Neurocirugía “Manuel Velasco Suárez”, 14269 Mexico City, Mexico

**Keywords:** Hydralazine, Valproic acid, iPSC, Fibroblasts, Reprogramming, Genes

## Abstract

**Background:**

The generation of induced pluripotent stem cells has opened the field of study for stem cell research, disease modeling and drug development. However, the epigenetic signatures present in somatic cells make cell reprogramming still an inefficient process. This epigenetic memory constitutes an obstacle in cellular reprogramming. Here, we report the effect of hydralazine (HYD) and valproic acid (VPA), two small molecules with proven epigenetic activity, on the expression of pluripotency genes in adult (aHF) and neonatal (nbHF) human fibroblasts.

**Methods:**

aHF and nbHF were treated with HYD and/or VPA, and viability and gene expression assays for OCT4, NANOG, c-MYC, KLF4, DNMT1, TET3, ARID1A and ARID2 by quantitative PCR were performed. aHF and nbHF were transfected with episomal plasmid bearing Yamanaka factors (OCT4, SOX2, KLF4 and c-MYC) and exposed to HYD and VPA to determine the reprogramming efficiency. Methylation sensitive restriction enzyme (MSRE) qPCR assays were performed on OCT4 and NANOG promoter regions. Immunofluorescence assays were carried out for pluripotency genes on iPSC derived from aHF and nbHF.

**Results:**

HYD upregulated the expression of OCT4 (2.5-fold) and NANOG (fourfold) genes but not c-Myc or KLF4 in aHF and had no significant effect on the expression of all these genes in nbHF. VPA upregulated the expression of NANOG (twofold) in aHF and c-MYC in nbHF, while it downregulated the expression of NANOG in nbHF. The combination of HYD and VPA canceled the OCT4 and NANOG overexpression induced by HYD in aHF, while it reinforced the effects of VPA on c-Myc expression in nbHF. The HYD-induced overexpression of OCT4 and NANOG in aHDF was not dependent on demethylation of gene promoters, and no changes in the reprogramming efficiency were observed in both cell populations despite the downregulation of epigenetic genes DNMT1, ARID1A, and ARID2 in nbHF.

**Conclusions:**

Our data provide evidence that HYD regulates the expression of OCT4 and NANOG pluripotency genes as well as ARID1A and ARID2 genes, two members of the SWI/SNF chromatin remodeling complex family, in normal human dermal fibroblasts.

**Supplementary Information:**

The online version contains supplementary material available at 10.1186/s13287-023-03268-w.

## Background

Induced pluripotent stem cells (iPSC) are derived from somatic cells that have been reprogrammed to an embryonic-like stage [1]. Somatic cell reprogramming is generated by the ectopic expression of genes associated with the regulation and maintenance of embryonic cells [2, 3]. Because the genomic sequences between the reprogrammed somatic cells and the generated iPSC do not have genetic differences, the reprogramming process is based on a reorganization of the cellular epigenome. The generation of iPSC involves the remodeling of the somatic epigenetic memory for the establishment of new epigenetic signatures similar to those found in pluripotent cells [4, 5]. However, one of the main obstacles during this process is the low reprogramming efficiency of somatic cells to iPSC. This low reprogramming efficiency is associated with the residual epigenetic memory of somatic cells that persists during and after the reprogramming process [6, 7]. Therefore, the search for small molecules that modify the structure of the epigenome and reactivate the expression of genes related to cellular reprogramming is of great interest.

Drugs with regulatory effects on the epigenome, termed “epigenetic drugs,” have been identified. Epigenetic drugs are mainly divided into two categories: the ones that modify DNA methylation patterns and those that inhibit histone deacetylases [8]. Within these two categories are hydralazine (HYD) and valproic acid (VPA). Hydralazine is a direct-acting peripheral vasodilator that acts primarily on the arteries, causing relaxation of smooth muscles [9]. HYD is indicated for the treatment of hypertensive disorders and heart failure; however, its current use is limited to hypertensive conditions during pregnancy [10]. The effect of HYD on the epigenome is related to changes in DNA methylation patterns by the inhibition of the DNMT1 enzyme [8, 11]. On the other hand, VPA is a short-chain fatty acid indicated for the treatment of epilepsy and bipolar disorder. VPA can act by increasing the levels of the neurotransmitter *γ*-aminobutyric acid (GABA) in the brain or by altering the properties of sodium channels [10, 12]. Nevertheless, the VPA mechanisms of its therapeutic action are not well-understood. VPA inhibits class I histone deacetylases, which generates a hyperacetylation of histones H3 and H4, causing changes in the chromatin structure that concludes in the transcriptional activation of several promoters [13, 14]. Therefore, in this work, we proposed the use of the epigenetic drugs HYD and VPA as a strategy to regulate the expression of pluripotency genes and to attempt to increase the reprogramming process in adult (aHF) and neonatal (nbHF) fibroblasts.

## Materials and methods

### Chemicals

HYD hydrochloride (purity 99%, catalog #H1753) and VPA sodium salt (purity 98%, catalog #P4543) were purchased from Sigma-Aldrich (St. Louis, MO, USA). For assays, HYD and VPA were diluted in culture medium from an aqueous stock solution.

### Cell culture, cell viability and IC_50_ values

Human adult dermal fibroblasts (aHF) (ATCC PCS-201–012) and neonatal foreskin BJ fibroblasts (nbHF) (ATCC CRL-2522), were cultured and cell viability was assessed according to conditions previously reported [15]. The IC_50_ values were determined with the dose–response curve of each drug at 72 h according to the GraphPad software method (log(inhibitor) vs. normalized response).

### RNA extraction, reverse transcription and quantitative PCR assays

For RNA extraction and reverse transcription, we followed the methods of Aguirre-Vázquez et al. [15]. The functionality of the cDNA was evaluated by amplification of r18S gene by PCR using the primers r18S-F 5′-GTTATTTCCAGCTCCAATAGCGTA-3′ and r18S-R 5′-GAACTACGACGGTATCTGATCGTC-3′. Quantitative PCR was performed as previously describe by Aguirre-Vazquez et al. (2021) [15]. The 7500 fast real-time PCR system (Applied Biosystem, Foster City, CA, USA) with TaqMan primers/probe assays for NANOG (Hs02387400_g1), OCT4 (POU5F1, Hs01895061_u1), MYC (Hs00153408_m1), KLF4 (Hs00358836_m1), TET3 (Hs00896441_m1), HIF1A (Hs00153153_m1), ARID1A (Hs00195664_m1) and ARID2 (Hs00326029_m1) was used. The PCR reaction was carried out in 20 μL with the TaqMan Universal PCR Master Mix (Applied Biosystem, Carlsbad, CA, USA). Amplification was performed in the standard mode under the following conditions: 50 °C for 2 min, followed by 95 °C for 10 min, and then 40 cycles at 95 °C for 15 s and 60 °C for 1 min. Quantification of DNMT1 gene expression was evaluated using primers F 5′-TACCTGGACGACCCTGACCTC-3′ and R 5′-CGTTGGCATCAAAGATGGACA-3′, as previously reported [16]. Following the qPCR reaction, a dissociation curve was generated to validate the specificity of the primers. Data were analyzed using the 2^−ΔΔCT^ [17] method. Human GAPDH (GAPDH) Endogenous Control (4310884E, Applied Biosystems) and/or Human TBP Endogenous Control (4326322E, Applied Biosystems) were performed in parallel with the TaqMan Gene Expression Assays, and for normalization of DNMT1, the following primers were used: GAPDH-F 5′-TTGGTATCGTGGAAGGACTCA-3′ and GAPDH-R 5′- TGTCATCATATTTGGCAGGTTT-3′. Technical triplicates of three biological replicates were considered for each experiment, where negative template controls were included for all assays.

### Methylation sensitive restriction enzyme (MSRE) qPCR

Genomic DNA from aHF and nbHF were extracted with Wizard Genomic DNA Purification kit (Promega) according to the manufacturer’s recommendations. Afterward, the analysis of DNA methylation with the EpiJET DNA Methylation Analysis Kit (*Msp*I/*Hpa*II) (Thermo Fisher Scientific, Vilnius, LT) according to the manufacturer’s instructions was performed. DNA digestion with *Msp*I and *Hpa*II enzymes or the undigested control reaction was carried out in 20 μL with 200 ng of gDNA for 4 h at 37 °C. Then, the samples were incubated at 90 °C for 10 min. Subsequently, qPCR was performed by technical duplicates from three biological replicates using 1 μL of digested (*Msp*I/*Hpa*II) or undigested genomic DNA in 10 μL volume using the EXPRESS SYBR GreenER qPCR SuperMix Universal (Invitrogen) and 0.35 nM of primer forward/reverse. The primer sequences used in this assay are shown in Table 1. Amplification was performed following the next reaction conditions: an initial incubation at 50 °C for 2 min, followed by 95 °C for 5 min, and then 40 cycles at 95 °C for 15 s, 63 °C for 30 s, and 72 °C for 30 s. Following the qPCR reaction, a dissociation curve was generated to validate the specificity of the primers. The percentage of 5-mC modification was calculated using the formula (2-Ct *Hpa*II Rx–Ct undigested Rx) × 100. Validation experiments were performed according to the manufacturer’s instructions.

**Table 1:** Primer sequences used for the MSRE-qPCR assays

Promoter region	Site	Sequence (5′-3′)	Amplicon size (bp)
OCT4	1	Forward CCT GCA CTG AGG TCC TGG A	81
		Reverse CCT AAT GGT GGT GGC AAT GGT	
	2	Forward GGG TTG AGC ACT TGT TTA GGG	112
		Reverse AGG TTC AAA GAA GCC TGG GAG	
	3	Forward CCC ACT GCC TTG TAG ACC TTC	124
		Reverse CCC ACT CTT ATG TTG CCT CTG T	
NANOG	1	Forward CCA CGG CCT CCC AAT TTA CTG	172
		Reverse ACC TGA AGA CAA ACC CAG CAA C	
	2	Forward CCT GAA GCA TGA TGT ACT AGC CC	186
		Reverse CTG GCT TTG CTC CCA CAC AAG	
	3	Forward GCG AAG AAT GTA GTA AGT CGG C	87
		Reverse CCA TTG TGT CTA GGG TAA GAG C	

### Generation of iPSC from aHF and nbHF

Reprogramming of aHF and nbHF was carried out with episomal reprogramming vectors pCXLE-hOCT3/4-shp53, pCLXE-hSK, and pCLXE-hUL [18]. Briefly, 1 × 10^6^ cells (between passages 6 and 10) were transfected with a 2.5 µg mix of each vector using a Neon Transfection System (Invitrogen). The conditions for aHF were 1800 V, 20 ms with one pulse, and for nbHF, they were 1650 V, 10 ms with three pulses. After transfection, cells were cultured for 7 days in reprogramming medium in the presence or absence of 30 µM HYD. The reprogramming medium was formulated with DMEM high glucose supplemented with 2.5 mM L-glutamine, 10% fetal bovine serum (Gibco), 10% KnockOut Serum Replacement (Gibco), 1 mM sodium pyruvate (Corning), 1% non-essential amino acids, 3 µM CHIR99021(Sigma-Aldrich) and 0.5 µM A83-01 (Sigma-Aldrich). At day 8, cells were recovered and seeded on mitotically inactivated mouse embryonic fibroblasts (iMEF). Medium was replaced to KnockOut DMEM supplemented with 20% KnockOut Serum Replacement, 2.5 mM Glutagro (Corning), 1% non-essential amino acids, 0.1 mM 2-mercaptoethanol, and 10 ng/mL of basic Fibroblast growth factor (bFGF) (Corning). Colonies were visualized and counted at 25–30 days, and those with characteristics of human ESC-like colonies [19, 20] were picked up for further experiments. Cultures were maintained in the conditions mentioned above.

### Immunofluorescence assays

iPSC colonies, aHF and nbHF, were cultured in 24-well plates with glass coverslips precoated with 0.5% gelatin according to the previously described culture conditions [15]. For the detection of the stage-specific embryonic antigen 4, SSEA4, permeabilization with Triton X-100 was omitted. Then, incubation with a primary antibody diluted in a blocking solution was performed overnight at 4 °C. After cell washing three times for 5 min, cells were incubated in the dark with appropriate secondary antibodies for 1 h at room temperature, counterstained with 4′,6-diamidino-2-phenylindole (DAPI), and mounted with SlowFade Diamond (Invitrogen). The following antibodies were used: goat anti-OCT4 (R&D Systems, AF1759, 1:100), mouse anti-SOX2 (R&D Systems, MAB2018, 1:100), rabbit anti-NANOG (Prepotech 500-P236, 1:1,000), mouse anti-SSEA4 (R&D Systems MAB1435, 1:100), rabbit anti-NRF2 (Abcam ab31163, 1:200) and HIF1A (Santa Cruz Biotechnology sc-13515, 1:25). The primary antibodies used for OCT4, SOX2 and SSEA4 detection were those included in the Human Pluripotent Stem Cell Marker Antibody Panel Plus (R&D Systems). The secondary antibodies goat anti-rabbit conjugated with Alexa Fluor 488, goat anti-mouse conjugated with Alexa Fluor 594, and donkey anti-goat conjugated with Alexa Fluor 568, all of Thermo Fisher Scientific, were used according to the provider’s instructions at 1:500 dilution. Immunostainings were analyzed and photographed with a resolution of 1,024 × 768 pixels using the EVOS microscope (Thermo Fisher Scientific, Bothell, WA, USA; serial number L0916-155G-0579) with 10x (AMG, 10X Plan FL, AMEP-4623) and 40x (AMG, 40X Plan FL, AMEP-4625) objective lenses coupled to the acquisition software EVOS FL Auto Cell Imaging System Software (Rev 26,059). Capture parameters were set initially at 50% brightness and 33% contrast for the three channels (DAPI, GFP and TxRed) and were adjusted depending on the signal intensity at 120 ms.

### Karyotyping

iPSC derived from aHF and nbHF were characterized by karyotyping through Laboratorios de Analisis Geneticos Especializados Mexico (LAGEM). G-banding in human metaphase chromosomes was analyzed in the ZEISS Axio Imager microscope (Carl Zeiss, Jena, Germany) using the Ikaros Karyotyping Software ver 5.9.0 (MetaSystems GmbH, Altlussheim, Germany) with an EC Epiplan Neofluar 100 × objective with correction to infinite coupled to the CoolCube 1—digital high -resolution progressive scan CCD camera (Metasystems), with 1360 × 1024 pixels of resolution.

### Statistics

Data are shown as the means values ± standard error of the mean. All data were analyzed by Mann–Whitney *U* test using the SPSS v2.0 software and GraphPad Prism 6 (San Diego, California, USA). The criterion for significance was set at *P* < 0.05 in all cases.

## Results

**Fig. 1:**
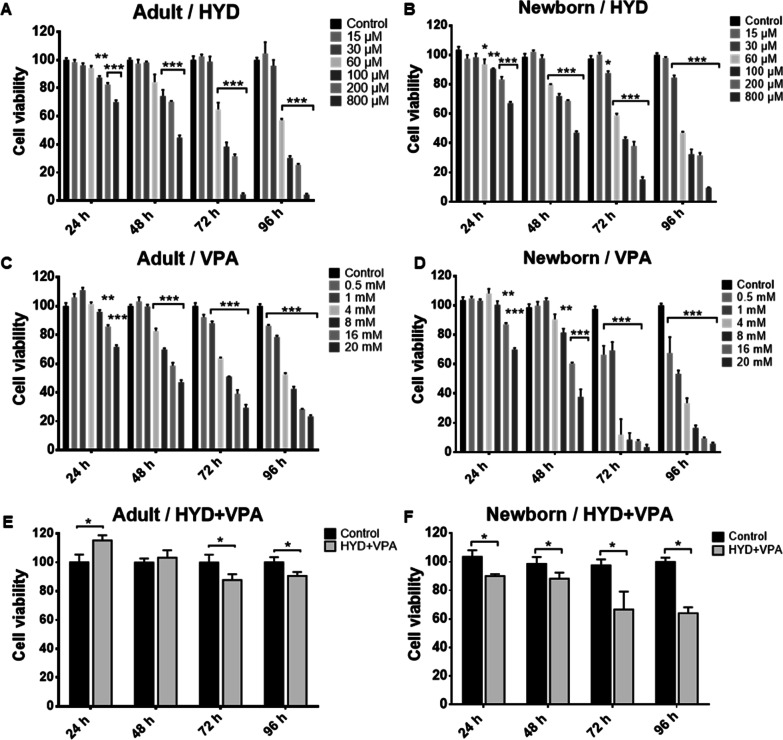
Effect of hydralazine (HYD) and valproic acid (VPA) on adult and neonatal fibroblast cell viability. Dose–time response curves were performed to evaluate the effect of HYD (panel **A** and **B**) and VPA (panel **C** and **D**) on adult and neonatal fibroblast cell viability. Two-way ANOVA with Dunnett multiple comparison tests was used for comparisons between control and other groups. The combined effect of 30 µM HYD and 1 mM VPA on cell viability of adult (panel **E**) and neonatal (panel **F**) fibroblasts during 96 h. The Mann–Whitney *U* test was used for comparisons between the control and HYD + VPA group. Values are expressed as mean ± standard error of the median from three independent experiments. *P < 0.05; **P < 0.01; ***P < 0.001

**Fig. 2:**
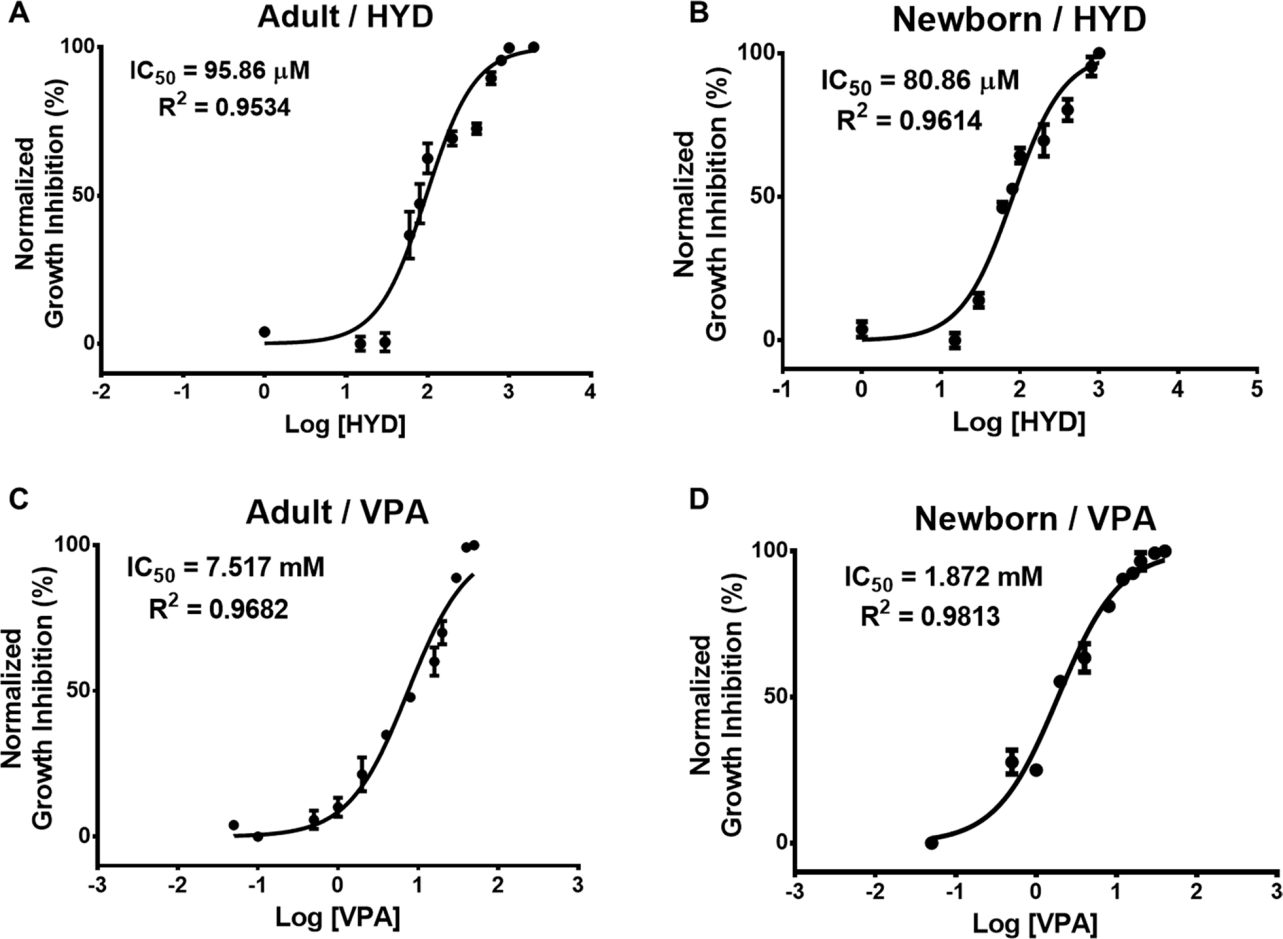
Half maximal inhibitory concentration (IC_50_) of hydralazine (HYD) and valproic acid (VPA) in adult and neonatal fibroblasts. IC_50_ values for HYD (panel **A** and **B**) and VPA (panel **C** and **D**) on adult and neonatal fibroblasts were determined at 72 h by dose–response curve fitting the log (inhibitor) vs. normalized response analytical method. Values are expressed as mean ± standard error of the median from three independent experiments. *R*^2^ values are displayed

To establish and validate a concentration of HYD and VPA that did not drastically reduce the viability of aHF and nbHF, dose–response time curves of the drugs were performed for a period of 24, 48, 72 and 96 h. A decrease in cell viability was observed that was related to the increase in concentration and the exposure time to HYD (Figs. 1A, B) and VPA (Figs. 1C, D) in both cell populations. According to the cell viability assays, 30 µM of HYD and 1 mM of VPA were selected for the following assays. Then, the combined effect of 30 µM of HYD and 1 mM of VPA (HYD-VPA) was evaluated on the cell viability of aHF and nbHF for 24, 48, 72 and 96 h. The cell viability of aHF only decreased by 17% (*P* < 0.05) and 15% (*P* < 0.05) at 72 and 96 h of HYD-VPA treatment, respectively (Fig. 1E). On the other hand, nbHF showed a decrease in cell viability of 11% (*P* < 0.05) from 24 h to just reaching 36% (*P* < 0.05) at 96 h (Fig. 1F). Our results indicate that the selected drug concentrations are suitable for the subsequent assays as they do not drastically decrease (no more than 50%) the viability of human fibroblasts. Furthermore, the viability curves allowed us to calculate the half-maximal inhibitory concentration (IC50) values for HYD and VPA at 72 h in both cell populations. For aHF, IC50 of 95.86 µM and 7.51 mM were calculated for HYD (Fig. 2A) and VPA (Fig. 2C), respectively. For nbHF, they showed a greater sensitivity to the drugs with IC_50_ values of 80.86 µM for HYD (Fig. 2B) and 1.87 mM for VPA (Fig. 2D).

**Fig. 3:**
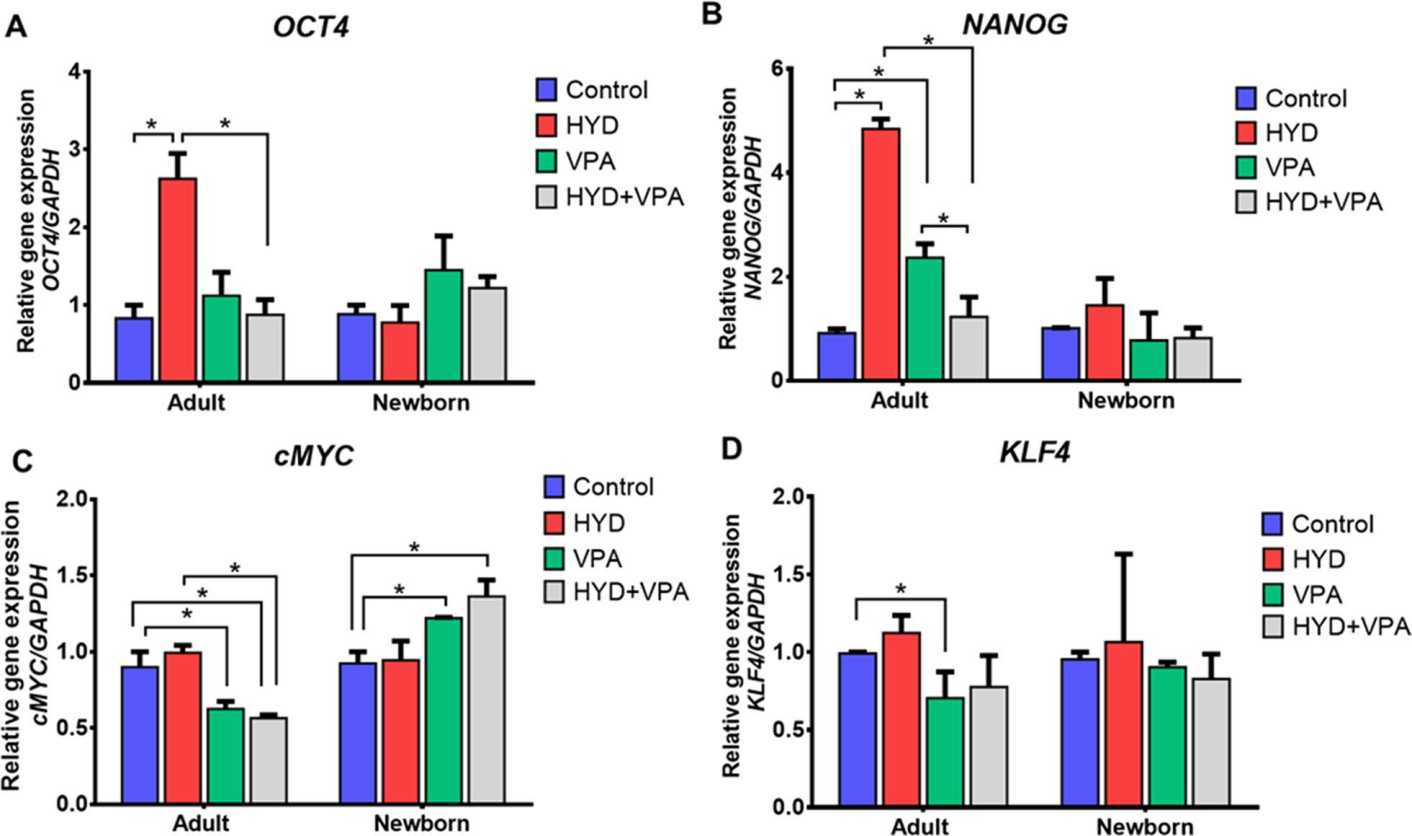
Expression of pluripotency genes by the effect of hydralazine (HYD) and valproic acid (VPA) in adult and neonatal fibroblasts. Adult and neonatal fibroblasts were treated for 72 h with 30 µM HYD, 1 mM VPA or the combination of both. Total RNA was extracted for each group, and RT-qPCR assays were performed for OCT4 (panel **A**), NANOG (panel **B**), c-Myc (panel **C**) and KLF4 (panel **D**) genes. Gene expression analysis were performed by technical triplicate of three biological replicates. Values are expressed as mean ± standard error of the median. The Mann–Whitney *U* test was used for comparisons between each group. *P < 0.05

Then, we analyzed whether the individual and the combined effect of the drugs modify the expression levels of the pluripotency genes in human somatic cells. To test this, aHF and nbHF were exposed to 30 µM HYD and/or 1 mM VPA for 72 h. Quantitative expression analysis of the OCT4 gene in HYD treated cells showed a threefold (*P* < 0.05) increase compared to the untreated group, although these changes were only seen in aHF (Fig. 3A). Interestingly, we observed that the HYD and VPA combination (HYD-VPA) nullifies the individual effect caused by HYD on OCT4 expression. Subsequently, our gene expression analysis of NANOG showed an increase of fivefold (*P* < 0.05) and twofold (*P* < 0.05) in the transcription levels by the individual effect of HYD and VPA in aHF, respectively (Fig. 3B). Analysis of c-Myc and KLF4 genes revealed a decrease in expression levels caused by the VPA (*P* < 0.05) and HYD-VPA (*P* < 0.05) treatments in aHF (Figs. 3C, D). In contrast, nbHF RT-qPCR assays showed an increase in c-Myc gene expression levels by the VPA (*P* < 0.05) and HYD-VPA treatments (Fig. 3D), and no significant changes in KLF4 expression were observed in these cells. Our results showed that the treatment with 30 µM HYD induced a significant expression of OCT4 and NANOG genes in adult fibroblasts.

**Fig. 4:**
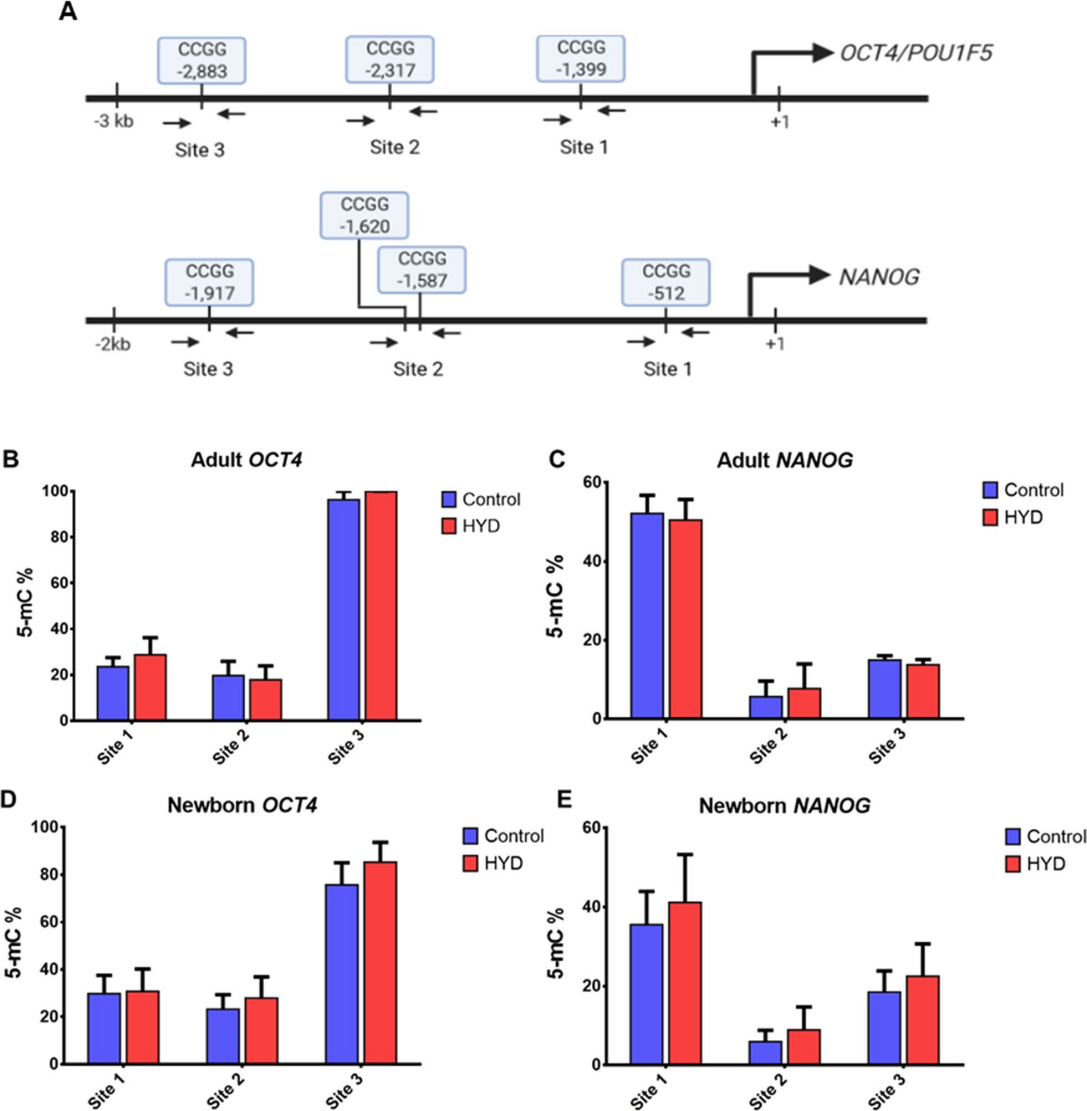
CpG methylation analysis of OCT4 and NANOG promoter regions in adult and neonatal fibroblasts. Panel **A**, schematic representation of CpG methylation (5′-CCGG-3′) sites at OCT4 and NANOG promoters. CpG methylation analysis of OCT4 (panel **B** and **D**) and NANOG (panel **C** and **E**) promoters in adult and neonatal fibroblasts. Fibroblasts were treated for 72 h with 30 µM HYD. Gene expression analysis were performed by technical duplicate of three biological replicates. Values are expressed as mean ± standard error of the median. The Mann–Whitney *U* test was used for comparisons between groups

To determine if the upregulation of OCT4 and NANOG expression by HYD was due to demethylation of promoter regions, we performed a methylation sensitive restriction enzyme qPCR (MSRE-qPCR) assay. Evaluation of 5-mC at three and four specific 5′-CCGG-3′ sites were performed in the proximal promoter region of OCT4 and NANOG, respectively (Fig. 4A). Contrary to expectations, our results did not show a decrease in the percentage of methylation in the promoter regions of OCT4 and NANOG genes of aHF (Figs. 4B, C). Likewise, nbHF showed an increasing trend in methylation percentage (Fig. 4D), particularly in the sites evaluated for the NANOG promoter (Fig. 4E). This effect may be a consequence of increased sensitivity to HYD of nbHF, but further studies are needed to define it. Together, the results showed that 30 µM HYD treatment for 72 h did not decrease DNA methylation in aHF and nbHF.

**Fig. 5:**
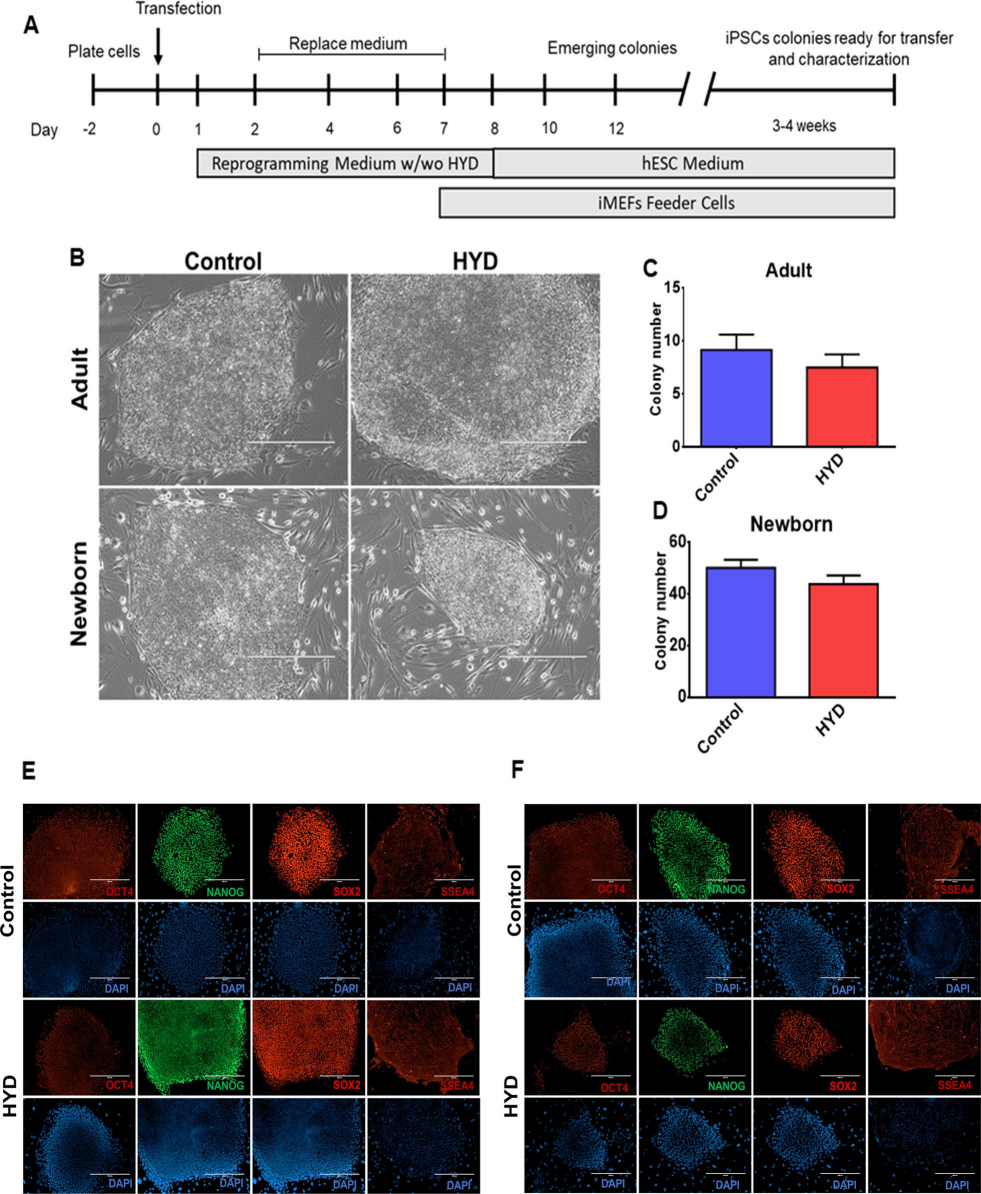
Evaluation of reprogramming efficiency by the effect of hydralazine (HYD) in adult and neonatal fibroblasts. Panel **A**, iPSC generation scheme with or without 30 µM HYD (w/wo HYD). hESC, human embryonic stem cells; iMEF, inactivated mouse embryonic fibroblasts. Panel **B**, representative images of the characteristic morphology of iPSC colonies (passage No. 3) from adult and neonatal fibroblasts. White bar in each micrograph corresponds to 400 µm. Colony number of iPSC with or without HYD treatment in adult (panel **C**) and neonatal (panel **D**) fibroblasts. Values are expressed as mean ± SEM from three independent experiments. Two-tailed Student's *t* test was used for comparisons between groups. Detection of pluripotency markers OCT4, NANOG, SOX2 and SEEA4 by immunofluorescence assays on iPSC colonies generated from adult (panel** E**) and neonatal (panel **F**) fibroblasts. Images were taken with a 10 × objective lens. White bar in each micrograph corresponds to 400 µm

Based on our quantitative expression assays, we asked if HYD treatment could enhance the reprogramming efficiency in aHF and nbHF after transfection with plasmids carrying the reprogramming factors [18], as assessed by colony formation and the presence of pluripotency-related proteins. To test this, a 30 µM HYD treatment scheme was designed in the initial stages of the reprogramming process (Fig. 5A). In both types of fibroblasts, the progressive formation of ESC-like phenotype colonies was observed. These ESC-like colonies were characterized by a high compaction degree, defined and rounded edges, and a large nucleus (Fig. 5B). To evaluate the reprogramming efficiency, the total number of colonies generated for both cell populations was counted. The selection criteria were based on the morphological characteristics of human pluripotent stem cell colonies previously described [19, 20]. Our results indicated that HYD did not increase the number of iPSC colonies in adult (Fig. 5C) and nbHF (Fig. 5D). Karyotyping displayed in iPSC derived from aHF (46, XX) and nbHF (46, XY), at passage 5 and 6, respectively, was normal (Additional file 1: Figure S1). Finally, the selected adult and neonatal iPSC colonies were characterized by the detection of the pluripotency markers OCT4, NANOG, SOX2, and the surface marker SSEA4 by immunofluorescence assays. All selected iPSC-like colonies were positive for the expression of pluripotency markers. These results confirmed that the reprogramming process was successfully achieved in aHF (Fig. 5E) and nbHF (Fig. 5F) fibroblasts in control colonies and those exposed to HYD. Taken together, these findings demonstrated that 30 µM HYD did not increase the number of iPSC colonies in aHF and nbHF.

**Fig. 6:**
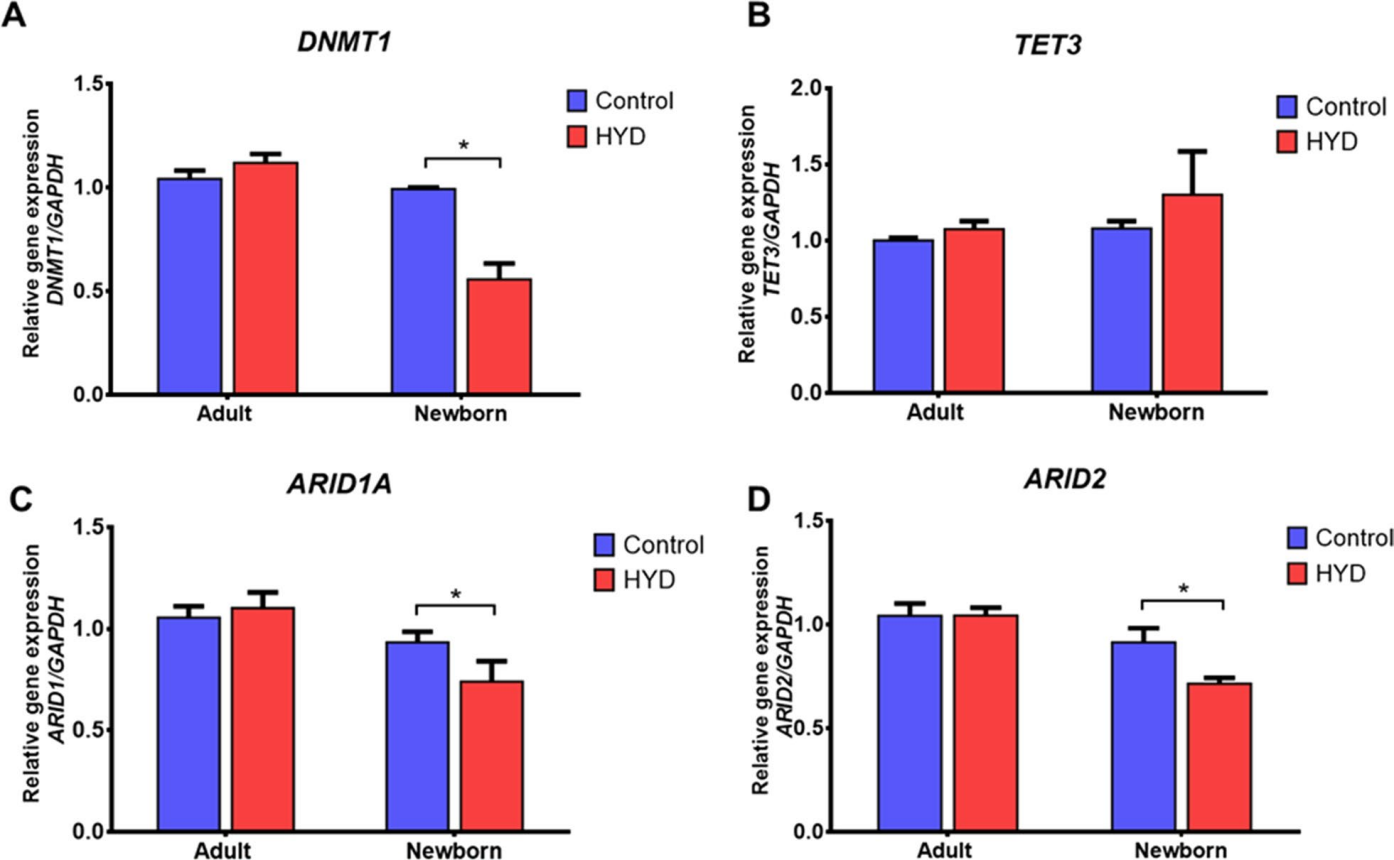
Expression analysis of genes implicated in DNA methylation and chromatin remodeling complexes by the effect of hydralazine (HYD). Adult and neonatal fibroblasts were treated for 72 h with 30 µM HYD. Total RNA was extracted for each group and RT-qPCR assays were performed for DNMT1 (panel **A**), TET3 (panel **B**), ARID1 (panel **C**), and ARID2 (panel **D**) genes. Gene expression analysis were performed by technical triplicate of three biological replicates. Values are expressed as mean ± SEM. The Mann–Whitney *U* test was used for comparisons between groups. *P < 0.05

Interestingly, it is well-known that HYD is a DNA methyltransferase 1 (DNMT1) inhibitor and that downregulation of DNMT1 activity improves the reprogramming efficiency; however, no changes in the number of colonies were observed. Therefore, to ascertain the effect of HYD as an epigenetic drug, we evaluated, by RT-qPCR assays, its effect on the expression of genes related to DNA methylation and genes involved in chromatin structure. First, we confirmed that HYD downregulated DNMT1 expression (*P* < 0.01), but this was only observed in nbHF (Fig. 6A). Then, we analyzed if the downregulation of DNMT1 enhances the expression of TET3, an enzyme related to active DNA demethylation. Although no significant changes were observed in both cell populations, an upregulation trend in nbHF was identified (Fig. 6B). Next, we decided to evaluate the expression levels of the ARID1A and ARID2 genes, both involved with the chromatin remodeling complex SWI/SNF. RT-qPCR analysis showed that the expression of ARID1A and ARID2 decreased 20% (*P* < 0.05) (Fig. 6C) and 21% (*P* < 0.05) (Fig. 6D) in nbHF, respectively. Our results confirm the effect of HYD as an inhibitor of DNMT1 and show, for the first time, that HYD is a transcriptional regulator of ARID1A and ARID2 genes.

## Discussion

**Fig. 7:**
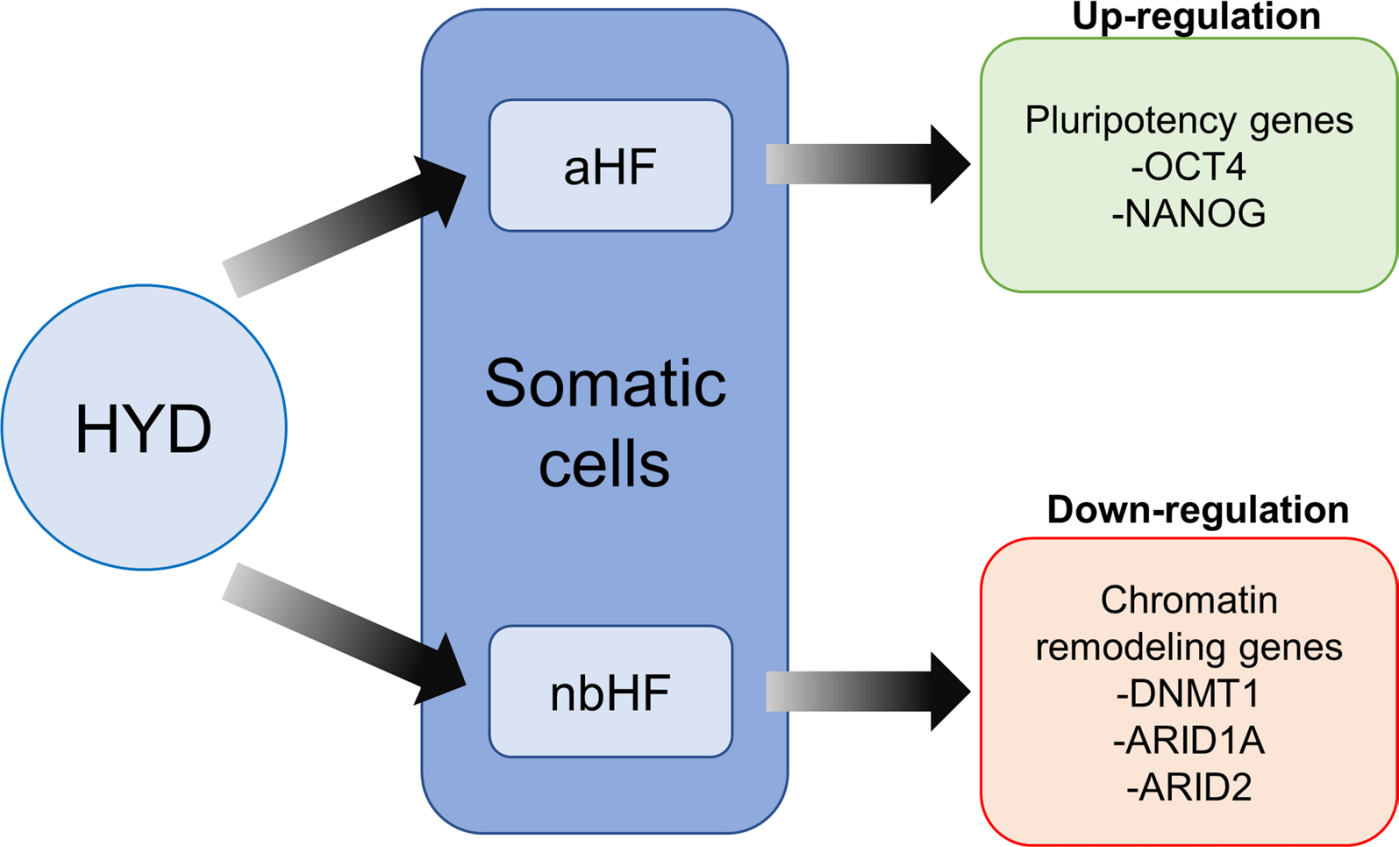
Schematic model of hydralazine (HYD) regulation on pluripotent and chromatin remodeling genes in human fibroblasts. HYD up-regulates OCT4 and NANOG genes in adult human fibroblasts (aHF) and down-regulates DNMT1, ARID1A and ARID2 genes in neonatal human fibroblasts (nbHF)

We have found that hydralazine modifies the expression of pluripotency genes in aHF and epigenetic genes in nbHF (Fig. 7), but it does not increase the number of iPSC colonies in both cell populations. Also, for the first time, we have discovered that HYD regulates the expression of ARID1 and ARID2 genes (Figs. 6C, D and 7), both as part of the chromatin remodeling complex SWI/SNF.

The transcriptional regulation of OCT4 and NANOG factors are related to the maintenance of the pluripotency network [21], oncogenesis [22], and cell reprogramming [23]. The reports related to the transcriptional regulation of OCT4 and NANOG genes in differentiated normal cells are limited. Our findings show that HYD increases the expression of OCT4 and NANOG genes in aHF at 72 h of treatment. On the contrary, O'Driscoll and colleagues reported that HYD downregulates the expression levels of OCT4 in pluripotent P19 cells [24]. The differences between the results are mainly attributed to the methodology used for the evaluation of OCT4 gene expression levels, the cellular model used for the assays, the HYD concentration, and the exposure time to the drug. First, the expression analysis performed in this work was determined by RT-qPCR, unlike the qualitative assay reported by O'Driscoll and colleagues. Likewise, our experimental scheme is focused on the treatment of normal human somatic cells, which is different from P19 cells derived from a mouse teratocarcinoma [24]. This is crucial because transcriptional regulation, epigenetics, and genome instability are different in P19 cells with pluripotent characteristics and somatic cells. In the other hand, there are other mechanisms, such as post-transcriptional regulation, that could be occurring in the evaluated genes that may explain the HYD effect on these types of cells. This hypothesis should be confirmed in further experiments.

Most reports assessing the effect of HYD on transcriptional regulation mainly focus on its repurposing or repositioning activity as adjuvant therapy in cancer treatments. Reactivation of tumor suppressor genes in hypermethylated promoter regions in cancer-derived cell lines has been related to HYD treatment [8, 25–27]. Likewise, HYD has been shown to reverse aberrant methylation in regulatory regions associated with renal fibrosis pathology [28]. Reactivation of gene expression due to HYD is mostly correlated with its epigenetic effect as a DNMT1 inhibitor [29, 30]. Our findings validate the inhibitory effect of HYD on the expression of the DNMT1 gene in nbHF (Figs. 6A and 7). However, our analyses of OCT4 and NANOG promoter regions do not show a correlation between the increase in the expression of pluripotency genes and a decrease in the evaluated CpG sites. Contrary to our expectations, we observed an increasing trend in the percentage of DNA methylation. It is important to consider that the MSRE-qPCR assay performed for the evaluation of CpG sites in the OCT4 and NANOG promoters is limited to the identification of the 5′-CCGG-3′ sequence recognized by *Msp*I and *Hpa*II enzymes. It is advisable to complement our analyses with techniques that allow to evaluate the total promoter region, such as bisulfite sequencing [31]. In addition, in somatic cells, OCT4 and NANOG genes are located in heterochromatin zones, and it is possible that 72 h of treatment with HYD is not enough to modify the methylation at the CpG sites of the evaluated promoter regions.

Our results indicate that the HYD-VPA combination annuls the individual effect of HYD on OCT4 and NANOG gene expression and downregulates the expression of the c-Myc gene in aHF. In this regard, HYD induced a lupus-like phenotype through the inhibition of the ERK pathway, causing downregulation of DNMT1 [30, 32]. Interestingly, reports have suggested that VPA is an ERK pathway activator in primary hepatocytes [33] and neural cells [34]. This leads us to presume that the function of each drug has an antagonistic effect, which is evident in their combined effect on pluripotency gene expression levels. On the other hand, this antagonistic effect of HYD and VPA does not explain the downregulation of c-Myc and KLF4 genes. In this regard, anti-proliferative and anti-metastatic effects in cancer-derived cell lines by the combination of HYD and VPA have been reported [10]. Additionally, the HYD-VPA combination decreases the expression levels of oncogenes and prometastatic genes in NIH 3T3-Ras cells. Pluripotency reprogramming and tumorigenesis share molecular mechanisms, such as oncogene activation, downregulation of tumor suppressor genes, epigenetic changes, and a metabolic switch [35]. This suggests that the changes generated in the expression of pluripotency genes by the combination HYD-VPA could be related to the anti-cancer effect of the drugs.

Although HYD did not increase the reprogramming efficiency in our experimental scheme, the use of small molecules capable of inhibiting DNMT1 activity has proven to be an effective strategy to enhance the reprogramming efficiency. Rodriguez-Madoz and colleagues showed that the reversible dual G9a/DNMT1 inhibitor molecule, CM272, enhances the mesenchymal to epithelial transition during the early phase of cell reprogramming [36]. Additionally, RG108, another small molecule DNMT1 inhibitor that has been used with other small molecules, increases the reprogramming efficiency [37]. These differences between HYD and the referenced small molecules could be related to the treatment schedule used during reprogramming, drug concentration, the reprogramming method, and the DNMT1 inhibition potency of each molecule.

Other epigenetic regulation mechanisms, independent of DNMT inhibition, have been described for HYD. Dehghan and colleagues reported the correlation between the activation of the histone deacetylase SIRT1 by hydralazine and stress resistance in C. elegans [38]. Likewise, Tampe and colleagues demonstrated that HYD-induced demethylation is mediated by active demethylation mechanisms, specifically by the methylcytosine dioxygenase TET3, ten-eleven translocation 3 protein, and not dependent on DNMT1 inhibition [28]. Our TET3 gene expression analyses showed an upward trend due to the effect of HYD. Furthermore, HYD treatment decreases the expression levels of ARID1A and ARID2 genes, both members of the SWI/SNF chromatin remodeling complex family. Interestingly, the decrease in ARID1A and ARID2 expression is related to epigenetic reprogramming and oncogenesis [39, 40]. This provides a new mechanism of epigenetic regulation mediated by hydralazine. Complementary studies are necessary to determine the effect of the reduction in the ARID1A and ARID2 transcription levels on the chromatin structure.

Although cell culture, treatments, and reprogramming assays were carried out under the same conditions, we observed a difference in the response to the evaluated drugs in both cell populations. We observed these differences in drug sensitivity assays, gene expression analyses, and the total number of iPSC colonies generated between aHF and nbHF. Interestingly, the activity of drug-metabolizing enzymes changes significantly from fetal to adult age [41, 42]. Therefore, we attribute these differences to regulatory changes related to the chronological age of each cell population used in this work. It might be relevant to extend the HYD and VPA effects to fibroblasts from other donors to confirm these findings.

Finally, we are aware of the limitations of our experimental strategy when evaluating pluripotency genes in human somatic cell lines. It is necessary to complement our expression analyses with the methodologies proposed by Li et al. [43] and Hou et al. [44]. In the former, the authors designed a luciferase assay system for identifying compounds that induce the expression of OCT4 and NANOG genes [43]. Likewise, Hou et al. generated transgenic mice expressing the GFP reporter gene under the control of the OCT4 promoter [44]. The application of both methodologies will make possible to confirm the effect observed in our expression assays. Finally, this work represents a first approach in the study of the effect of HYD and VPA on the expression of pluripotency genes in human somatic cells.

## Conclusions

In this study, we demonstrate that HYD modifies the expression of groups of genes involved in the induction of pluripotency and chromatin remodeling in aHF and nbHF. HYD and VPA have limited effects on the transcriptional regulation of pluripotency genes, which have basal expression levels in our cell models. For this reason, we believe that the effect of both drugs should be evaluated in multipotent, pluripotent stem cells or fibroblasts from other donors to explore whether the effects observed in the gene expression, reprogramming, and epigenetic assays described here occur in other cell models. The main perspective of this work is that treatment with HYD, alone or in combination with other epigenetic modulators, is a promising option to induce the expression of pluripotency genes and chromatin remodeling complexes. Further studies are needed to explore the effect of hydralazine on epigenetic signatures such as acetylation, histone methylation, and the global evaluation of methylated regions in DNA.

## Supplementary Information

**Additional file1**. **Figure S1**. Chromosomal analysis of iPSC derived from aHF and nbHF.Karyotyping was carried out using standard G-banding in metaphase chromosomes.Analysis revealed normal karyotypes in aHF (Panel **A**, 46,XY) and nbHF (panel **B**,46,XY) at passage 5 and 6, respectively.

## Data Availability

The data presented in this study are available on request from the corresponding author.

## Abbreviations

DAPI: 4′,6-diamidino-2-phenylindole
aHF: Adult human fibroblasts
bFGF: Basic fibroblast growth factor
DNMT1: DNA methyltransferase 1
GABA: *γ*-Aminobutyric acid
HYD: Hydralazine
gDNA: Genomic deoxyribonucleic acid
IC_50_: Half-maximal inhibitory concentration
iMEF: Inactivated mouse embryonic fibroblasts
iPSC: Induced pluripotent stem cells
M-MLV: Moloney murine leukemia virus
MSRE: Methylation sensitive restriction enzyme
PCR: Polymerase chain reaction
qPCR: Quantitative polymerase chain reaction
nbHF: Neonatal human fibroblasts
PBS: Phosphate-buffered saline
TBP: TATA-box binding protein
VPA: Valproic acid
