# Supplementary figures and images for: Cell-type dependent regulation of pluripotency and chromatin remodeling genes by hydralazine
Source: Stem Cell Res Ther. 2023 Mar 16;14:42. doi: 10.1186/s13287-023-03268-w (PMC10021945; doi:10.1186/s13287-023-03268-w)

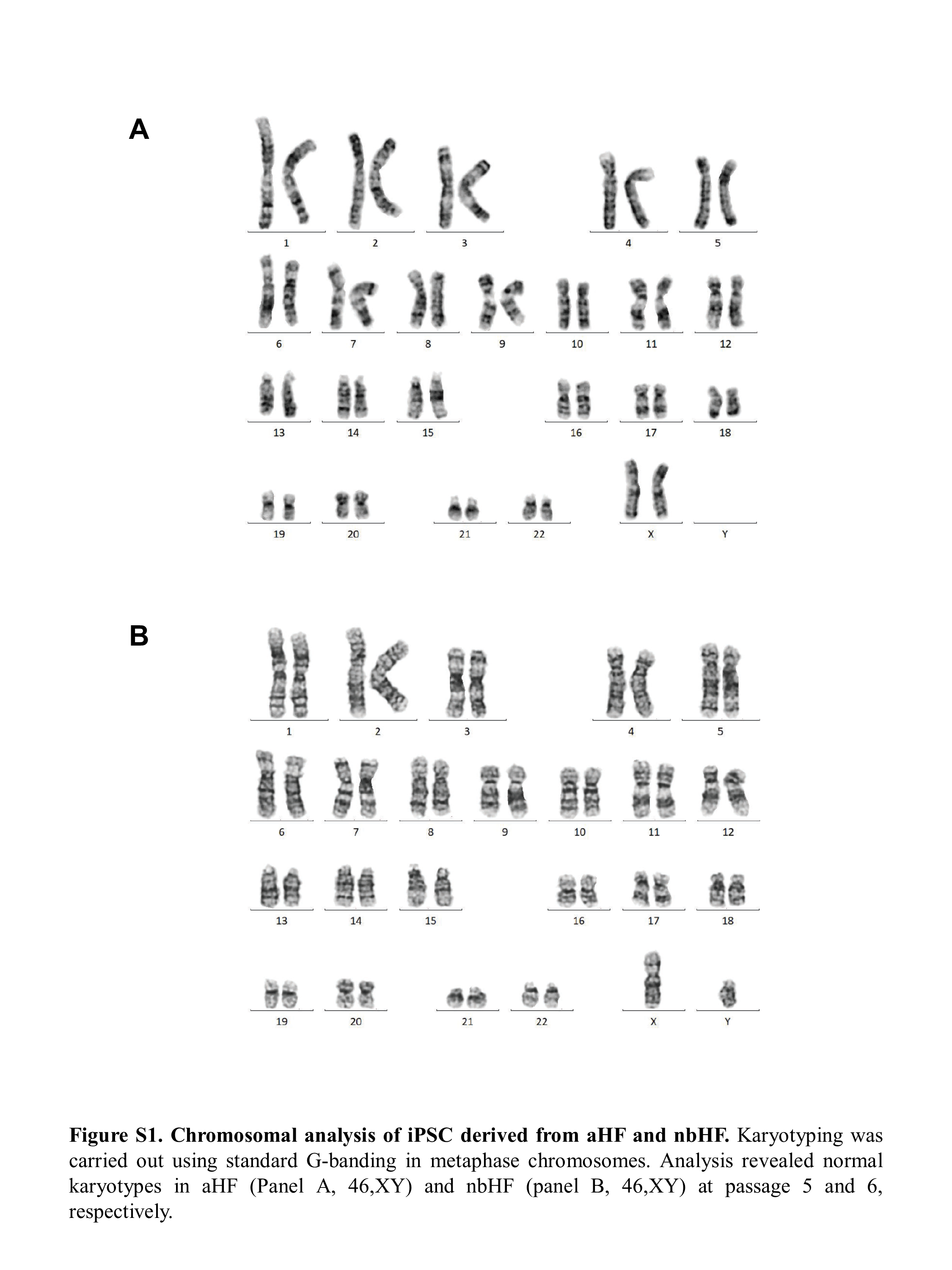

Supplement: Supplementary file 1 — Additional file1. Figure S1. Chromosomal analysis of iPSC derived from aHF and nbHF.Karyotyping was carried out using standard G-banding in metaphase chromosomes.Analysis revealed normal karyotypes in aHF (Panel A, 46,XY) and nbHF (panel B,46,XY) at passage 5 and 6, respectively. [file 13287_2023_3268_MOESM1_ESM.tif]
